# The mitochondrial energy metabolism pathway-related signature predicts prognosis and indicates immune microenvironment infiltration in osteosarcoma

**DOI:** 10.1097/MD.0000000000036046

**Published:** 2023-11-17

**Authors:** Sen Yang, Liyun Liu, Xiaoyun Liu, Xinghua Li, Yuyu Zheng, Zeen Ren, Ruijiang Wang, Yun Wang, Qian Li

**Affiliations:** a Department of Orthopedics, The Peace Hospital of Changzhi City, The First Clinical Hospital of Changzhi Medical University, Changzhi, Shanxi Province, China; b Department of General Medical, The People’s Hospital of Changzhi City, The Third Clinical Hospital of Changzhi Medical University, Changzhi, Shanxi Province, China; c Department of Orthopedics, The Second People’s Hospital of Changzhi City, The Fourth Clinical Hospital of Changzhi Medical University, Changzhi, Shanxi Province, China; d School of Basic Medicine, Medical College of Baicheng City, Baicheng, Jilin Province, China.

**Keywords:** gene signature, mitochondrial energy metabolism, osteosarcoma, overall survival, risk score

## Abstract

**Background:**

Abnormalities in the mitochondrial energy metabolism pathways are closely related to the occurrence and development of many cancers. Furthermore, abnormal genes in mitochondrial energy metabolism pathways may be novel targets and biomarkers for the diagnosis and treatment of osteosarcoma. In this study, we aimed to establish a mitochondrial energy metabolism-related gene signature for osteosarcoma prognosis.

**Methods:**

We first obtained differentially expressed genes based on the metastatic status of 84 patients with osteosarcoma from the TARGET database. After Venn analysis of differentially expressed genes and mitochondrial energy metabolism pathway-related genes (MMRGs), 2 key genes were obtained using univariate Cox regression and least absolute shrinkage and selection operator (LASSO) regression analysis. Next, we used these 2 genes to establish a prognostic signature. Subsequent analyses elucidated the correlation between these 2 key genes with clinical features and 28 types of immune cells. Pathway changes in osteosarcoma pathogenesis under different metastatic states were clarified using gene set enrichment analysis (GSEA) of differentially expressed genes.

**Results:**

A gene signature composed of 2 key prognosis-related genes (KCNJ5 and PFKFB2) was identified. A risk score was calculated based on the gene signature, which divided osteosarcoma patients into low- or high-risk groups that showed good and poor prognosis, respectively. High expression of these 2 key genes is associated with low-risk group in patients with osteosarcoma. We constructed an accurate nomogram to help clinicians assess the survival time of patients with osteosarcoma. The results of immune cell infiltration level showed that the high-risk group had lower levels of immune cell infiltration. GSEA revealed changes in immune regulation and hypoxia stress pathways in osteosarcoma under different metastatic states.

**Conclusion:**

Our study identified an excellent gene signature that could be helpful in improving the prognosis of patients with osteosarcoma.

## 1. Introduction

Osteosarcoma is a primary malignant bone tumor that most commonly affects children, adolescents, and young adults.^[1]^ It was reported approximately 900 new cases of osteosarcoma diagnosed every year in the United States, accounting for <0.2% of all cancers and 20% to 40% of all bone cancers.^[2,3]^ It tends to occur in the metaphysis of the long bones, with poor prognosis and high disability rates. Lung metastasis directly reduces the survival of patients with osteosarcoma.^[4,5]^ Although standard treatment concepts have emerged, includes neoadjuvant chemotherapy with doxorubicin, methotrexate and cisplatin followed by surgical resection of the primary tumor and adjuvant chemotherapy,^[6]^ osteosarcoma patients without lung metastases have a 70% 5-year survival rate, which decreases to 20% in patients with lung metastases.^[7]^ Therefore, clarifying the specific mechanism of osteosarcoma remains the basis for effective intervention in progression and metastasis.

Mitochondria are organelles that provide energy; they not only maintain the biological functions of cells, but also regulate oncogenic signaling, innate immunity, and apoptosis. Mitochondrial energy metabolism can regulate stem cell functions through many mechanisms, including glycolysis, redox reactions in oxidative phosphorylation, energy metabolism process conversion, changes in mitochondrial membrane potential, production of intracellular reactive oxygen species, and oxidative stress.^[8,9]^ Emerging evidence indicates that mitochondrial metabolic pathways are reprogrammed in cancer and play vital roles in bioenergetics, biosynthesis, and redox homeostasis.^[10]^ One of the most well-known abnormal metabolic characteristics of cancer cells is the Warburg effect, a state of highly elevated glucose uptake and a preference for glycolysis rather than mitochondrial oxidative phosphorylation for ATP production, even in the presence of sufficient oxygen.^[11]^ Moderate increases in the mitochondrial metabolite reactive oxygen species and overdecomposition of glutamine play a positive role in promoting cancer cell proliferation, tumor growth, and metastasis.^[12,13]^ Based on the above theories, we believe that abnormal mitochondria-mediated energy metabolism plays a role in the occurrence, development, and metastasis of osteosarcoma. Continued research on mitochondrial metabolic reprogramming and its role in maintaining redox homeostasis is crucial for identifying future cancer therapies.^[10]^ A deeper understanding of the mechanism of mitochondria-mediated energy metabolism in osteosarcoma may lead to the development of novel treatments for osteosarcoma. Hence, we conducted this study to identify key mitochondrial energy metabolism pathway-related genes (MMRGs) and to construct an accurate prognostic mitochondrial energy metabolism-related gene signature of patients with osteosarcoma from TARGET database. The identified mitochondrial energy metabolism-related gene signature may supplement the current predictors of osteosarcoma prognosis and help understand the relationship between mitochondrial energy metabolism and osteosarcoma progression.

## 2. Materials and Methods

### 2.1. Acquisition of osteosarcoma datasets and screening of MMRGs

RNA-seq data and relevant clinical data for 84 patients with osteosarcoma were downloaded from the TARGET database using UCSC XENA. Mitochondrial energy metabolism-related gene data were downloaded from the literature^[14]^ and the GeneCard database. The MMRGs are listed in Table S1, http://links.lww.com/MD/K676.

Patients with osteosarcoma were divided into 2 groups according to metastasis, with 63 and 21 patients in the metastatic and non-metastatic groups, respectively. We used the “DESeq2” package to analyze differentially expressed genes (DEGs) between the metastatic and non-metastatic groups. A |log2-fold change| >0.5 and *P* value < .05 were used to screen DEGs. The 612 DEGs obtained in the above steps and 382 MMRGs were analyzed using a Venn diagram. Ten MMRGs were identified for downstream analysis. A gene expression heatmap of the 10 MMRGs was drawn by the “ComplexHeatmap” package for R software (version 3.6.3). Functional enrichment analysis and visualization of the 10 MMRGs were performed using the “clusterProfiler,” “org.Hs.e.g..db,” and “GOplot” packages.

### 2.2. Screening of prognosis-related genes and construction of the prognostic model

Univariate Cox regression analysis was performed to identify prognosis-related genes in osteosarcoma. A prognostic model was constructed based on the 10 MMRGs. We also identified prognostic genes by LASSO regression analysis using R software. After Venn analysis of the LASSO regression and univariate COX results, we obtained the prognosis-related genes related to mitochondrial energy metabolism and visualized them on the chromosome using R software.

The risk scoring system was established based on the following formula:


$$\begin{array}{l} Risk\;score=\overset{n}{\underset{i=1}{\sum }}expr_{genei}\times coefficient_{genei}. \end{array}$$

A risk factor plot was constructed using the “ggplot2” package. The “time ROC” package was used to draw receiver operating characteristic (ROC) curves. The 84 patients with osteosarcoma were divided into high- and low-risk groups according to the median risk score. The “survminer” package was used to generate survival curves.

### 2.3. Prognosis-related gene expression changes under different clinical characteristics.

We analyzed expression differences of selected prognosis-related genes in different clinical characteristics by using the “ggplot2,” “stats” and “car” packages.

### 2.4. Construction and evaluation of the nomogram

A nomogram was constructed using selected prognosis-related genes as independent prognostic parameters to predict the probability of overall survival (OS) at 1 year, 3 years, and 5 years by the “rms” package. The discriminative ability of the nomogram was verified through ROC and calibration analyses.

### 2.5. Gene set enrichment analysis (GSEA)

R software was used to identify DEGs between the high- and low-risk groups of osteosarcoma patients. The “clusterProfler” package was used for the GSEA. The “ggplot2” package was used for visualization.

### 2.6. Immune cell infiltration level analysis

We used single-sample gene set enrichment analysis (ssGSEA) to calculate the immune infiltration landscapes of 84 osteosarcoma patients. The ssGSEA method is a machine-learning algorithm used to characterize intratumoral immune landscapes and can identify the expression of 28 types of immune cells. We compared the infiltration of 28 immune cells between the high- and low-risk groups. Furthermore, we analyzed the relationship between the abundance of 28 immune cells and the expression of selected prognosis-related genes.

### 2.7. Statistical analysis

R software (version 3.6.3) was used to acquire statistical data. Prognosis-related genes were identified using univariate Cox regression and LASSO regression analyses. We constructed a prognostic model based on the selected prognosis-related genes. The prediction efficiency of the prognostic model was assessed using a time-dependent ROC analysis. Student *t* test was used to evaluate the significance of differences. Statistical significance was set at *P* value < .05.

## 3. Results

### 3.1. Identification of differentially expressed MMRGs and functional annotation.

**Figure 1.**
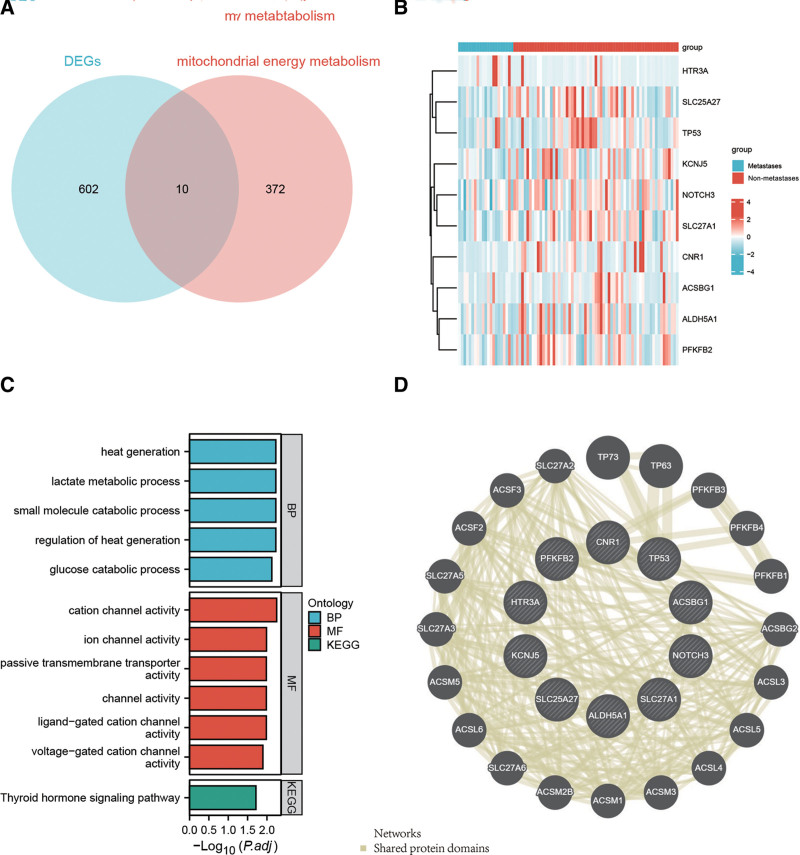
Identification and functional enrichment analysis of MMRGs between Osteosarcoma patients with metastasis and non-metastasis group. (A) Venn analysis of the intersection between MMRGs and DEGs identified. (B) Heatmap visualizing the expression of 10 DEGs related to the mitochondrial energy metabolism pathway in the 2 subgroups. (C) Terms of Gene Ontology (GO) enrichment analysis and KEGG pathways related to the 10 MMRGs. (D) PPI network construction of differentially expressed MMRGs. DEGs = differentially expressed genes, KEGG = Kyoto encyclopedia of genes and genomes, MMRGs = mitochondrial energy metabolism pathway-related genes, PPI = protein–protein interaction.

As shown in Figure 1A and B, 10 DEGs related to the mitochondrial energy metabolism pathway were identified using a Venn diagram and visualized using a heatmap. Functional annotation analysis of differentially expressed MMRGs is important for understanding the mechanisms of mitochondrial energy metabolism in osteosarcoma. Therefore, we performed gene ontology (GO) and Kyoto Encyclopedia of Genes and Genomes analyses. The GO analysis results showed that the differentially expressed MMRGs were mainly involved in response to heat generation (BP), lactate metabolic process (BP), small molecule catabolic process (BP), regulation of heat generation (BP), cation channel activity (MF), ion channel activity (MF), passive transmembrane transporter activity (MF), channel activity () and ligand-gated cation channel activity (MF). Kyoto encyclopedia of genes and genomes analysis identified the thyroid hormone signaling pathway (Fig. 1C). All results showed that the energy metabolism function of osteosarcoma changes significantly in different metastatic states. In addition, to better understand the potential biological function of differentially expressed MMRGs in osteosarcoma, we performed Metascape protein–protein interaction enrichment analysis (Fig. 1D).

### 3.2. Construction of a prognostic mitochondrial energy metabolism-related gene signature

**Figure 2.**
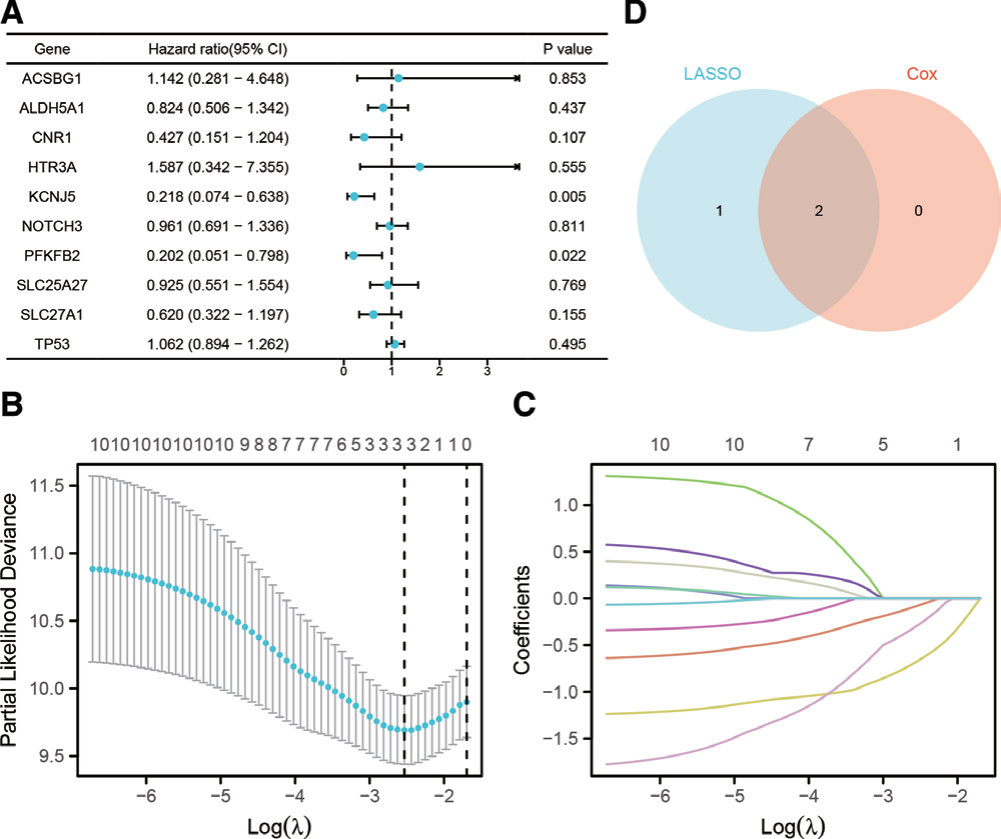
Construction of a risk prognostic model based on 10 MMRGs. (A) Univariate Cox regression analysis was performed for all prognosis-related genes. The value of *P* < .05 was considerate statistically significant. (B) The LASSO regression model of the 10 MMRGs performed by Lasso-ten-fold cross-validation. (C) Cross-validation for tuning the parameter selection in the LASSO regression. (D) Venn diagram of the intersection between the LASSO regression model and Univariate Cox regression analysis. LASSO = least absolute shrinkage and selection operator, MMRGs = mitochondrial energy metabolism pathway-related genes.

We selected key prognosis-related genes from 10 differentially expressed MMRGs based on univariate Cox regression and LASSO regression analyses to construct an accurate prognostic signature. First, we performed preliminary dimensionality reduction for 10 differentially expressed MMRGs through univariate Cox regression analysis, and 2 genes (KCNJ5 and PFKFB2) with *P* value < .05 were selected (Fig. 2A). LASSO regression analysis identified 3 prognostic genes (Fig. 2B and C, Table S2, http://links.lww.com/MD/K677), which were used to construct the prognostic model. In accordance with this prognostic model, each patient was assigned a risk score and grouped into a high- or low-risk group depending on the score. Finally, after Venn analysis of the genes obtained using the above 2 methods (Fig. 2D), 2 key prognosis-related genes (KCNJ5 and PFKFB2) were identified (Table 1).

**Table 1:** Description of key prognosis-related genes.

Gene symbol	Description	Log2fc	*P* value
KCNJ5	G protein-activated inward rectifier potassium channel 4	-0.863219848	.018323049
PFKFB2	6-phosphofructo-2-kinase/fructose-2,6-bisphosphatase 2	-0.578353603	.031981406

### 3.3. Chromosomal mapping of prognosis-related genes

**Figure 3.**
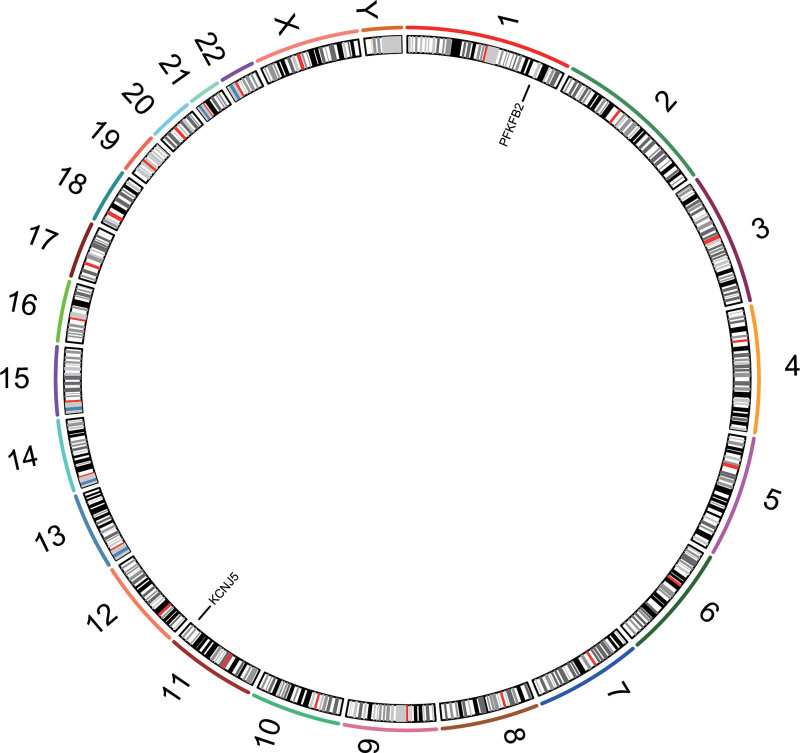
Chromosomal mapping of 2 prognosis-related genes.

The selected prognosis-related genes were visualized using chromosomal mapping. As shown in Figure 3, KCNJ5 is located on chromosome 11 and PFKFB2 on chromosome 1.

### 3.4. Analysis of the constructed prognostic model

**Figure 4.**
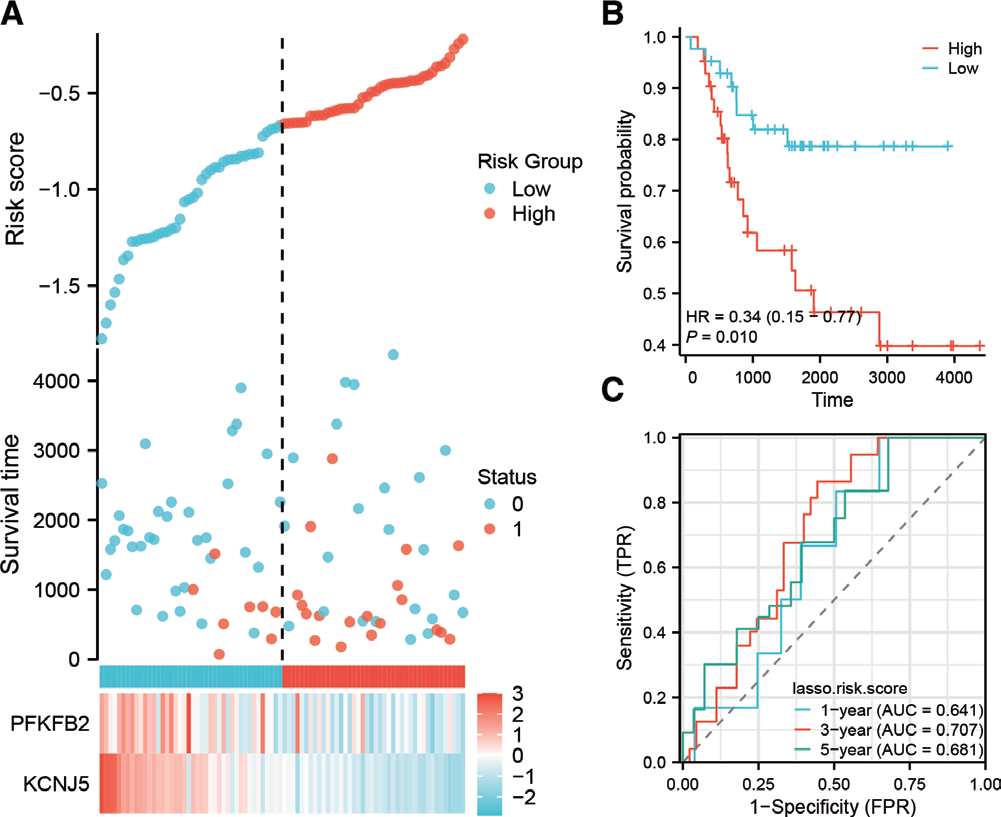
Construction and validation of risk score model in 84 Osteosarcoma patients. (A) The risk score, survival time distributions and gene expression heat map of MMRGs in the Osteosarcoma patients. (B) The ROC curves of the risk scoring model predicting overall survival of 1-yr, 3-yr, and 5-yr. (C) Kaplan–Meier curves showing the overall survival of patients in the high-risk and low-risk groups. MMRGs = mitochondrial energy metabolism pathway-related genes, ROC = receiver operating characteristic.

As shown in Figure 4A, patients were divided into 2 subgroups based on their risk score calculated using the constructed prognostic model, and the expression levels of the 2 key prognosis-related genes used for model construction are presented in a heatmap. Survival analysis indicated that patients in the low-risk group had better prognosis than those in the high-risk group (Fig. 4B). ROC analysis indicated 1-year, 3-years, and 5-years AUCs of 0.641, 0.707, and 0.681, respectively (Fig. 4C). The value of AUCs is >0.5 and the closer it is to 1, the better the prediction performance. These results demonstrated that the constructed prognostic model was effective and stable.

### 3.5. The correlation between prognostic genes and clinical characteristics

**Figure 5.**
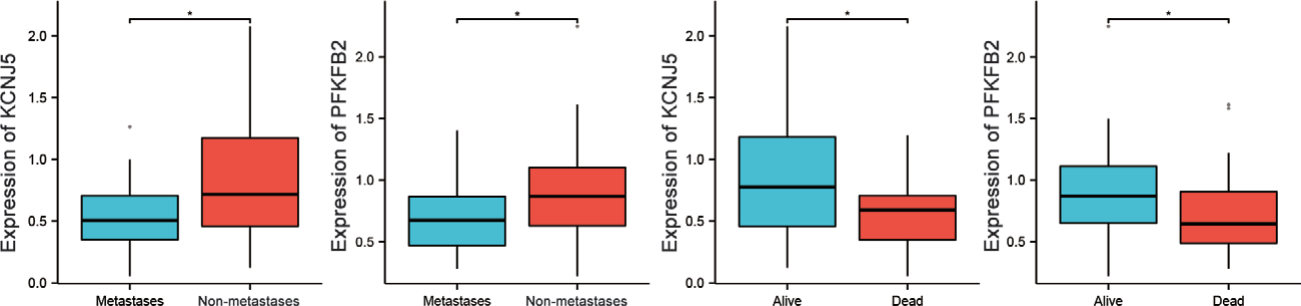
Expression of KCNJ5 and PFKFB2 in different survival and metastases states.

As shown in Figure 5, we observed changes in the expression of KCNJ5 and PFKFB2 in different survival and metastatic states. The expression levels of these 2 key prognosis-related genes were significantly higher in those who survived and did not have metastasis than in those who died and did have metastasis.

### 3.6. Construction and validation of a nomogram based on KCNJ5 and PFKFB2

**Figure 6.**
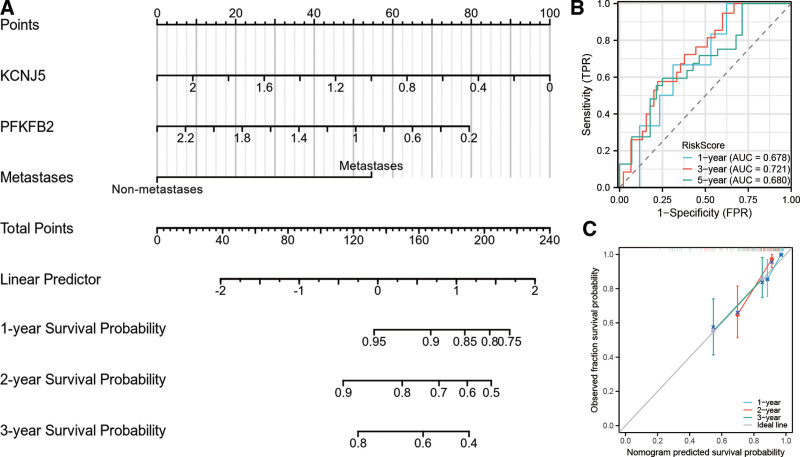
Prognostic nomogram for the 1-yr, 3-yr, and 5-yr OS of osteosarcoma patients. (A) The independent risk factors that affect the OS of osteosarcoma patients were incorporated into the nomogram model. (B) The ROC curves for predicting the nomogram of 1-yr, 3-yr, and 5-yr OS. (C) The nomogram calibration curves for predicting the 1-yr, 3-yr, and 5-yr OS. OS = overall survival, ROC = receiver operating characteristic.

To better predict the survival rate of patients with osteosarcoma, we constructed a nomogram that integrated the prognostic model and clinical characteristics (metastasis condition). As shown in Figure 6A, the total score for each item was added to predict the survival rates within 1, 3, and 5 years. We then calculated the score of each osteosarcoma patient based on the nomogram and evaluated the predictive ability of the nomogram through ROC analysis. The nomogram AUCs for the 1-year, 3-years, and 5-years OS rates were 0.678, 0.721, and 0.680, respectively (Fig. 6B). The calibration curves of this nomogram showed a high consistency between the observed and predicted values (Fig. 6C).

### 3.7. GSEA

**Figure 7.**
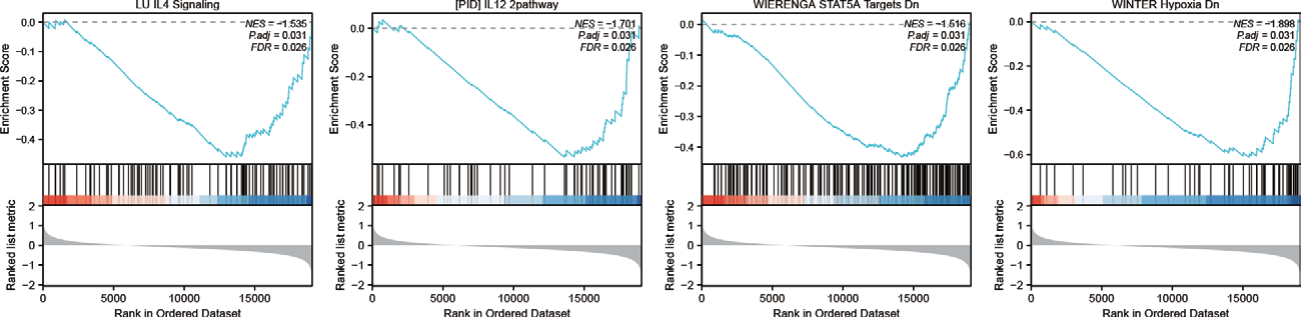
Enrichment plots from GSEA. Several pathways and biological processes were differentially enriched in the osteosarcoma patients. (A) LU IL4 Signaling. (B) PID_IL12_2PATHWAY. (C) WIERENGA STAT5A Targets Dn. (D) WINTER Hypoxia Dn. GSEA = gene set enrichment analysis.

To reveal the potential impact of MMRGs on the occurrence and development of osteosarcoma, we performed GSEA of DEGs between the metastatic and non-metastatic groups. The GSEA results showed enrichment of many pathways, including the LU-IL4-Signaling, (PID) IL12-2 Pathway, WIERENGA-STAT5A-Targets-Dn, and WINTER-Hypoxia Dn (Fig.7).

### 3.8. Immune cell infiltration level analysis

**Figure 8.**
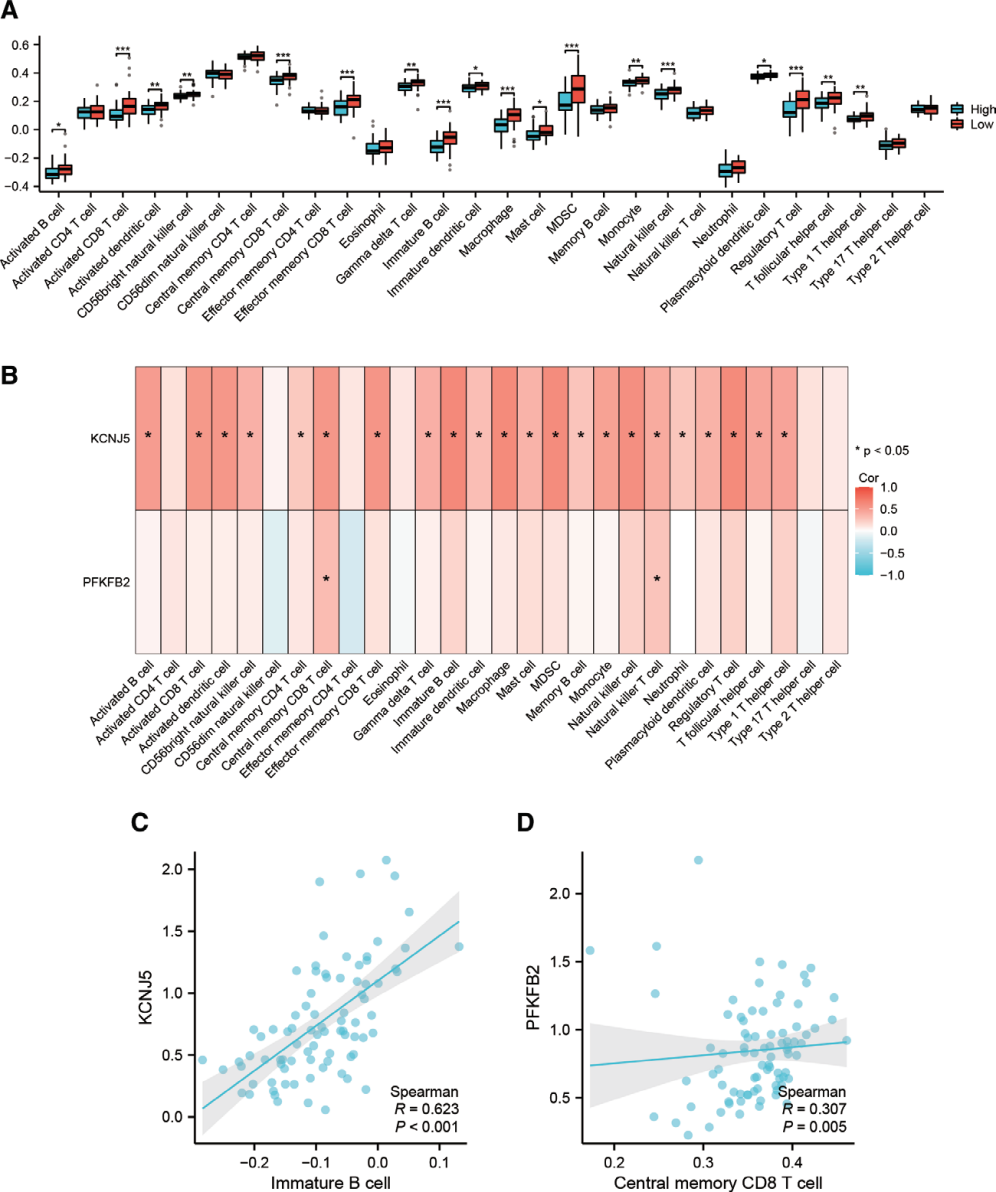
Assessing the potential of the gene signature in immune cell infiltration level. (A) Difference in 28 kinds of immune cell infiltration between high- and low-risk group (*P < .05; **P < .01; ***P < .001). (B) The correlation between 28 kinds of immune cells and 2 key genes. (C) Correlation analysis between KCNJ5 expression and immature B cells. (D) Correlation analysis between PFKFB2 expression and central memory CD8 T cells.

The ssGSEA method was used to first determine the infiltration of 28 different immune cell types in osteosarcoma, and then Spearman analysis was used to examine the relationship between the 2 key prognosis-related genes and immune cell infiltration. The results showed that the high-risk group had lower levels of immune cell infiltration, including activated CD8 T cells (*P* < .001), activated dendritic cells (*P* < .01), central memory CD8 T cells (*P* < .001), effector memory CD8 T cells (*P* < .001), immature B cells (*P* < .01), natural killer cells (*P* < .001), and type 1 helper cells (*P* < .01) (Fig. 8A). Moreover, we analyzed the correlation between KCNJ5, PFKFB2, and immune cells using a heat map (Fig. 8B). The expression of KCNJ5 and PFKFB2 correlated positively with immature cells (*R* = 0.623) and central memory CD8 T cells (*R* = 0.307), respectively (Fig. 8C and D).

## 4. Discussion

Despite many current treatment methods for osteosarcoma, including surgery, radiotherapy, chemotherapy, and neoadjuvant chemotherapy, the effect is not good owing to its high invasiveness, metastasis, and recurrence characteristics. In addition, the tolerance of osteosarcoma to traditional chemotherapy drugs leads to poor treatment outcomes. Drugs that promote tumor cell death and inhibit angiogenesis have become popular research topics in tumor treatment. Therefore, a profound understanding of the specific pathogenesis of osteosarcoma and the discovery of important target genes can provide new ideas for effective early treatment and reliable clinical prognosis.

Mitochondria are bioenergetic and biosynthetic organelles that take up substrates from the cytoplasm and use them to drive fatty acid oxidation, the TCA cycle, the electron transport chain, and respiration, and to synthesize amino acids, lipids, nucleotides, heme, and iron sulfur clusters, as well as NADPH for mitochondrial antioxidant defense.^[15]^ Mitochondrial pathway abnormalities and metabolic disorders can lead to changes in gene expression that promote cancer development, progression, and immune system evasion.^[16]^ Therefore, we believe that the abnormality of mitochondrial energy metabolism and its related key genes play an important role in the occurrence of osteosarcoma.

In this study, we used 2 step-by-step analysis methods (univariate Cox regression analysis and LASSO regression analysis) to identify 2 key prognosis-related genes (KCNJ5 and PFKFB2) and used the identified genes to construct a gene signature. KCNJ5 is a Potassium Inwardly Rectifying Channel Subfamily J Member 5, which encodes a G-protein-activated inward rectifier potassium channel.^[17]^ This potassium channel mediates the outward current of potassium ions to maintain hyperpolarization and stabilize the resting membrane potential.^[18]^ To date, most studies have focused on the relationship between the KCNJ5 mutation and aldosteronism caused by adrenal adenoma,^[19]^ and it has been found that overexpression of KCNJ5 can inhibit the growth of tumor cells.^[20,21]^ In a study on gastric cancer, the inhibition of oxytocin signaling that promotes cancer significantly increased the expression of KCNJ5.^[22]^ In our analysis, the expression of KCNJ5 was higher in osteosarcoma patients who did not have metastasis and survived than in those who had metastasis and died. The bifunctional 6-phosphofructo-2-kinase (PFKFB) enzyme family was recently identified to contribute to the Warburg effect.^[23,24]^ Among the 4 isozymes (PFKFB1–4) in this family, PFKFB2 has been found to play a key role in regulating tumor growth and survival in multiple cancer types, including gastric cancer, gliomas, and osteosarcoma.^[25–30]^ It has been found that expression of PFKFB2 can be regulated by miR-1297 and inhibit the proliferation and aerobic glycolysis of osteosarcoma cells.^[31]^ Activation of the SRC/ERK/c-MYC/PFKFB2 pathway plays a cancer-promoting role in osteosarcoma.^[32]^ However, in our study, the expression of PFKFB2 showed the same trend as that of KCNJ5, which was significantly higher in patients without metastasis and in those who survived. This may be because overexpression of PFKFB2 inhibits the progression of osteosarcoma, although the specific mechanism needs to be further studied.

To better predict the prognosis of patients with osteosarcoma, we constructed a nomogram integrating their clinical characteristics, including metastatic status. In this nomogram, the risk score based on the gene signature had the highest weighted score, followed by the metastasis condition. The prognostic prediction efficiency of the nomogram was confirmed by ROC analysis in terms of 1-y, 3-y and 5-y survival rates.

Moreover, the GSEA results indicated that these mitochondrial energy metabolism-related genes may play a potential role in the regulation of the immune system, tumor cell apoptosis, and hypoxia. IL-4 affects osteosarcoma growth through humoral immunity.^[33]^ IL-12 upregulates Fas expression in tumor cells and increases immune-mediated destruction of osteosarcoma cells.^[34]^ Studies have found that decreased STAT5A expression predicts poor prognosis in osteosarcoma.^[35]^ The rapid growth and insufficient nutrient supply of solid tumors result in tumor hypoxia,^[36]^ which can promote tumor angiogenesis and metastasis.^[37]^ The above studies confirm the reliability of our findings, but the specific mechanism of each pathway in osteosarcoma still needs to be explored.

Immune and stromal cells are major components of the tumor microenvironment.^[38,39]^ Tumors have several immunosuppressive mechanisms, such as increased immunosuppressive cells (regulatory T cells and tumor-associated macrophages), decreased expression of cancer antigens, and increased expression of immune checkpoints (CTL4A and PD-1).^[40,41]^ In our study, high-risk osteosarcoma patients generally had lower levels of immune cell infiltration, and the risk score was negatively correlated with infiltrating immune cells. One study claimed that CD8 + T cells might be associated with good prognosis.^[42]^ Li et al^[43]^ analyzed immune cells in the microenvironment of osteosarcoma, Ewing sarcoma, multiple myeloma, and cancer bone metastases and found that osteosarcoma patients with high infiltration of B cells had a better prognosis and that activated B cells correlated positively with survival. Yang et al^[44]^ conducted a comprehensive analysis of immune infiltration in the osteosarcoma microenvironment and concluded that male patients had more NK cells than female patients did. The above studies all proposed the effect of immune infiltrating cells on osteosarcoma through the study of certain immune cells. Our study systematically evaluated the infiltration of immune cells in osteosarcoma through MMRGs, providing new ideas and methods for studying immune infiltration in osteosarcoma.

There were some limitations to our study. First, owing to the limited clinical data provided by public datasets, we were unable to incorporate a sufficient number of clinical characteristics, which may result in potential biases in the prediction of survival time. Second, owing to limited experimental conditions and difficulty in accessing clinical samples, the key prognosis-related genes in this study could not be verified experimentally. Third, a single microarray analysis may contribute to a high false-positive rate and one-sided results; thus, it is necessary to improve detection power by integrating multiple individual data in a future study. Despite these limitations, this study adds to our understanding of the 2 key MMRGs that can effectively predict the prognosis of osteosarcoma.

## 5. Conclusions

In summary, we offer new insights into the association between mitochondrial energy metabolism and osteosarcoma. We explored mitochondrial energy metabolism-related gene expression and its prognostic implications in osteosarcoma and identified a mitochondrial energy metabolism-related gene signature to establish a risk model with good prognostic prediction performance. Simultaneously, we built a nomogram to predict the survival probabilities of patients with osteosarcoma, and the calibration curves showed good predictive ability. Therefore, our study will help to improve the prognosis of patients with osteosarcoma.

## Acknowledgments

We are grateful to the TARGET database for providing the platform and to the contributors for uploading their meaningful datasets.

## Author contributions

**Data curation:** Sen Yang.

**Investigation:** Xiaoyun Liu, Ruijiang Wang, Qian Li.

**Methodology:** Zeen Ren, Qian Li.

**Project administration:** Xinghua Li, Yuyu Zheng.

**Software:** Xiaoyun Liu, Yun Wang.

**Supervision:** Liyun Liu.

**Writing – original draft:** Sen Yang.

**Writing – review & editing:** Sen Yang.

## Supplementary Material




