# Supplementary material for: The mitochondrial energy metabolism pathway-related signature predicts prognosis and indicates immune microenvironment infiltration in osteosarcoma
Source: Medicine (Baltimore). 2023 Nov 17;102(46):e36046. doi: 10.1097/MD.0000000000036046 (PMC10659617; doi:10.1097/MD.0000000000036046)
Supplement: Supplementary file 1 [file medi-102-e36046-s001.pdf]

mitochondrial energy metabolism

ACADL
ALDH18A1
ADH1B
PPAT
GAPDH
ALDH2
ACSL4
PPARG
CYP2U1
PFKP
ACSL1
ADH1A
GPI
ALDH3B2
NDUFS6
CYP4A22-AS1
PC
MDH2
CYC1
PFKFB2
ACAT1
CPT1B
ACAA2
CYP4A26P
NDUFB4
ACAA1
MDH1B
NDUFS8
NDUFAB1
ALDH3B1
ATP4A
COX5B
PFKP-DT
ACSBG1
COX6C
NDUFB9
ALDH1B1
ALDH1A2
NDUFA9
CYP4A11
NDUFB11
NDUFA12
PFKFB4
IDH1
ACADSB
ATP4B
ALDH3A1
PAAF1
ACADS
PPAN
SDHC
MDH1
PGM2

PFKFB3
NDUFB5
ALDH1L2
PKLR
NDUFA8
HADHA
NDUFB3
ALDH1L1
CPT1A
CYP4A22
CYP4A27P
ADH1C
EHHADH
NDUFB10
OXCT2
OXCT1
ACADM
ALDH4A1
PDHB
NDUFA7
COX7B
UQCRFS1
ACLY
PFKL
IDH3G
ACOX2
NDUFA10
PFKFB1
GAPDHS
ECI1
COX10
ALDH8A1
ALDH6A1
NDUFA11
CYP4A44P
ALDH9A1
ADH4
NDUFA6
MINPP1
AKR1A1
ACO1
NDUFV2
NDUFA13
NDUFA1
PDC
ADPGK
IDH3B
PPATP1
ATP12A
ECI2
CPT2
AHR
MDH1P2
CYP4A43P

HMGCL
POR
GCK
DLST
COX17
HADHB
ACOX3
ALDH1L1-AS1
ALDH3A2
LHPP
PPARA
ACSL6
PPARD
ACSBG2
NDUFB7
ALDH5A1
HADH
NDUFS7
ACSL6-AS1
ALDH1A1
PPA2
NDUFA4
COX15
PFKM
NDUFC2
BPGM
ADH6
NDUFV3
ACSL3
ACAT2
ALDH16A1
SDHD
NDUFB2
ACSL3-AS1
NDUFB6
NDUFC1
ALDH7A1P2
COX7C
GALM
ALDH7A1P4
PPATP2
ALDH7A1
ALDH1A3
ACSL5
PPAN-P2RY11
ALDH7A1P3
NDUFB8
ALDH1L1-AS2
ALDH7A1P1
ACO2
OXCT1-AS1
COX11
OXCT2P1
NDUFA2

NDUFA3
NDUFB1
PPA1
MDH1P1
CS
COX5A
PPARGC1A
SDHB
OGDH
DLAT
NDUFV1
NDUFS4
ECHS1
NDUFS1
NDUFS2
ACADVL
GCDH
PPARGC1B
DLD
SDHA
ACOX1
IDH3A
NDUFS3
PDHX
CPT1C
NDUFS5
NDUFA5
C1QBP
GFM2
TIGAR
HTT
NFATC4
LIAS
TANGO2
ETFA
AK2
CYCS
FBXL4
SLC25A20
COQ9
SLC25A3
ETFB
PTEN
PDP1
UCP2
LDHA
EIF4E
PRKCZ
RPS6KB1
KNG1
POLG
EIF4EBP1
ACAD9
KLK4

CR1
TNNT1
CD200
OMA1
TP53
SOD1
TLR2
CNR1
SCO2
UCP1
AK3
UCP3
TCF19
DNAJC19
SUCLA2
TUFM
GFM1
AUH
HIBCH
TSFM
TAFAZZIN
MTIF2
TMEM70
OPA3
SERAC1
CENPO
MTIF3
LIX1
C2orf88
NFU1
LIPT2
FH
LIPT1
IBA57
SRC
SIRT3
ESRRA
TPK1
AGK
MFF
AIFM1
BDNF
NR1H4
CRAT
NGLY1
DHTKD1
GPBAR1
CHCHD10
PKHD1
UCHL1
GSK3B
ASS1
PAH
PRKN

ATP7B
IRS1
KCNK9
MFN2
SCN9A
SOD2
TPI1
VDAC1
BTD
OCRL
ATP5F1B
RARS2
SUMF1
KRIT1
MIPEP
SLC27A1
SZT2
MITD1
FAM210B
MT-ND4
ESR1
PSEN1
CAT
NOTCH3
ADK
APOE
CBS
FOXO1
STIM1
CASP7
GLS
PGK1
SLC25A4
YWHAE
ADORA2B
EIF2AK3
HPRT1
HSPD1
HTR2A
PKD2
HSPA9
IL1B
INS
KCNJ5
RYR1
VCL
ADORA2A
HTR3A
PRDX6
RYR2
UQCRC2
YWHAZ
COX4I1
FXN

HK2
HUWE1
NEU1
SHMT1
SIRT5
XBP1
BAD
BSG
CKB
CLOCK
FUS
GDAP1
ID2
IGF2BP2
SLC25A5
ATG7
DBT
EIF2S1
ESRRG
HTR2B
NME4
PDK4
PNPLA6
PROK2
PTPA
SOAT2
TFAM
UQCRC1
EPO
LRPPRC
DMRT1
FLVCR2
NDUFAF1
OLA1
RYR3
SIRT4
SMPD2
SSBP1
SURF1
BICD2
GLRX5
IMMT
INF2
MAVS
ACAD10
ALYREF
ATP5PF
COX7A2L
MRS2
NDUFAF3
BOLA3
SLC25A27
TIMMDC1
ATP5MC1

FAHD1
OCIAD1
MT-ATP6
MT-CO1
MT-ND1
