# Supplementary material for: The mitochondrial energy metabolism pathway-related signature predicts prognosis and indicates immune microenvironment infiltration in osteosarcoma
Source: Medicine (Baltimore). 2023 Nov 17;102(46):e36046. doi: 10.1097/MD.0000000000036046 (PMC10659617; doi:10.1097/MD.0000000000036046)
Supplement: Supplementary file 2 [file medi-102-e36046-s002.pdf]

LASSO
CNR1
KCNJ5
PFKFB2
